# Clinical genomic profiling of malignant giant cell tumor of bone: A retrospective analysis using a real‑world database

**DOI:** 10.3892/mi.2024.141

**Published:** 2024-02-22

**Authors:** Yusuke Tsuda, Koichi Okajima, Yuki Ishibashi, Liuzhe Zhang, Toshihide Hirai, Hidenori Kage, Aya Shinozaki-Ushiku, Katsutoshi Oda, Sakae Tanaka, Hiroshi Kobayashi

**Affiliations:** 1 Department of Orthopedic Surgery, The University of Tokyo Hospital, Tokyo 113-8655, Japan; 2 Department of Oral and Maxillofacial Surgery, The University of Tokyo Hospital, Tokyo 113-8655, Japan; 3 Next-Generation Precision Medicine Development Laboratory, The University of Tokyo Hospital, Tokyo 113-8655, Japan; 4 Department of Respiratory Medicine, The University of Tokyo Hospital, Tokyo 113-8655, Japan; 5 Division of Integrative Genomics, The University of Tokyo, Tokyo 113-8655, Japan; 6 Department of Gynecology, The University of Tokyo Hospital, Tokyo 113-8655, Japan

**Keywords:** malignant giant cell tumor of bone, MAPK signaling, sarcoma, genome

## Abstract

Malignant giant cell tumor of bone (GCTB) is identified by the presence of multinucleated giant cells, with an aggressive behavior and a high risk of metastasis, which has not been genetically characterized in detail. H3 histone family member 3A (*H3F3A*) gene mutations are highly recurrent and specific in GCTB. The present study analyzed the clinical information and genomic sequencing data of eight cases of malignant GCTB (out of 384 bone sarcoma samples) using an anonymized genomic database. There were 5 males and 3 females among the cases, with a median age of 33 years at the time of the initial diagnosis. H3F3A G34W and G34L mutations were detected in 3 patients and 1 patient, respectively. In 75% of cases without *H3F3A* mutation, mitogen-activated protein kinase (MAPK) signaling pathway gene alterations were found (KRAS single nucleotide variant, KRAS amplification, nuclear respiratory factor 1*-BRAF* fusion). Moreover, the collagen type I alpha 2 chain*-ALK* fusion was detected in remaining one case. The most frequent gene alterations were related to cell cycle regulators, including *TP53*, *RB1*, cyclin-dependent kinase inhibitor 2A/B and cyclin E1 (75%, 6 of 8 cases). On the whole, the present study discovered recurrent MAPK signaling gene alterations or other gene alterations in cases of malignant GCTB. Of note, two fusion genes should be carefully validated following the pathology re-review by sarcoma pathologists. These two fusion genes may be detected in resembling tumors, which contain giant cells, apart from malignant GCTB. The real-world data used herein provide a unique perspective on genomic alterations in clinicopathologically diagnosed malignant GCTB.

## Introduction

Malignant giant cell tumor of bone (GCTB) is a clinicopathologically defined diagnostic concept characterized by the presence of multinucleated giant cells and an aggressive clinical behavior associated with a high risk of metastasis or local recurrence (1). Malignant GCTB is treated by wide resection; however, the prognosis is unfavorable (2).

H3 histone family member 3A (*H3F3A*) encodes for a H3.3 protein. GCTB is genetically characterized by a highly recurrent mutation in *H3F3A*, with the G34W mutation being the most common (1-3). The H3.3 G34W mutation is highly specific for GCTB, and almost all histological mimics lack this genetic signature (4,5). The loss of H3.3K36me3 on mutant H 3.3 alters the deposition of the repressive H3K27me3 mark from intergenic to genic regions, beyond areas of H3.3 deposition. This alteration promotes the redistribution of other chromatin marks and aberrant transcription, altering cell fate in mesenchymal progenitors and hindering differentiation (6). Previous studies have reported that the *H3F3A* mutations can also be detected in malignant GCTB (5,7). However, some malignant GCTBs have been found to be negative for *H3F3A* mutations, even though the paired GCTB component has been found positive for *H3F3A* mutations (5). Other reports suggested that *TP53* mutation, *KRAS/HRAS* mutation, *TERT* mutation, *KDM4B/KDM6A* loss, or H3K27me3 loss may be associated with the malignant progression of GCTB (8-11). However, oncogenic events in *H3F3A* wild-type malignant GCTB remain unknown.

In the present study, it was hypothesized that as-yet-unknown molecular events participate in the progression of malignant GCTB. Therefore, the present study analyzed genomic alterations in 8 cases of clinicopathologically diagnosed malignant GCTB using the Center for Cancer Genomics and Advanced Therapeutics (C-CAT) genomic database.

## Patients and methods

### Study design

The present study retrospectively analyzed the results of genomic profiling tests using extracted data from a Japanese nationwide genomic database (C-CAT).

### Comprehensive genomic profiling and the C-CAT database

In Japan, insurance coverage for the cancer comprehensive genomic profiling (CGP) test was implemented in June, 2019 (12,13). In total, three types of CGP tests are available through the national health insurance system for patients with advanced solid tumors who have completed standard chemotherapy or for whom no appropriate standard chemotherapy is available: The Foundation One^®^ CDx (F1CDx; Foundation Medicine, Inc.) test, Foundation One^®^ Liquid CDx (F1LCDx; Foundation Medicine, Inc.) test and the OncoGuide NCC Oncopanel System (https://www.ncc.go.jp/en/information/press_release/20190717/20190717152024.html). C-CAT information is available elsewhere (13). Briefly, C-CAT was established at the National Cancer Center as an organization that collects and facilitates the use of data derived from CGP tests (12,13). C-CAT collects CGP results and clinical information for almost all patients undergoing CGP after obtaining written informed consent. These data can be used in clinical trials and drug development following approval by both the institutional review board and C-CAT. As of March, 2023, >50,000 patients with advanced-stage cancer have undergone CGP tests since June, 2019.

### Data extraction

A search was made on the anonymized C-CAT database of genomic and clinical information on patients with malignant bone tumors. The clinical data in C-CAT include age, sex, histology, treatment before and after CGP tests, drug response and type of CGP test used. A total of 384 samples of genomic data were detected in the malignant bone tumor cohort of C-CAT from 2019 to 2022. Of these, eight malignant GCTB datasets were extracted for the present study. In other words, the genomic data of sequencing analysis results were already available and actual sequencing or mutation analysis was not performed during the present study. All eight samples were sequenced by F1CDx. Information on gene alterations was annotated using Cancer Knowledge Databases, such as OncoKB, ClinVar and COSMIC, etc, at C-CAT (13).

The F1CDx assay employs formalin-fixed paraffin-embedded tumor tissue samples obtained via biopsy or surgical procedure, with pathologists selecting suitable tumor specimens for testing (details available at https://www.foundationmedicine.com/genomic-testing/foundation-one-cdx). All histological diagnoses were made using morphology, immunohistochemistry and molecular data by specialized clinicians and pathologists in each hospital. The present study was approved by the Institutional Review Board of the University of Tokyo (Tokyo, Japan; approval no. 2021341G) and the C-CAT information utilization review committee (proposal control no. CDU2022-026 N).

### Statistical analysis

A Student's t-test test was used to compare the quantitative variables between two groups. A two-tailed probability (P)-value <0.05 was considered to indicate a statistically significant difference. Statistical analyses were performed using SPSS version 22.0 software (IBM Corp.).

## Results

### Clinical characteristics

The clinical characteristics of the 8 patients with malignant GCTB whose data were analyzed in the present study are summarized in Table I. The median age of the patients was 33 years, and 5 patients (63%) were male. A total of seven samples were collected from the primary sites, and one sample was collected from a metastatic lesion. Of the 8 patients included, 5 (63%) patients had metastasis, including to the lung, bone, peritoneum, spinal cord, soft tissue, or adrenal gland, when the F1CDx test was performed. A total of 5 patients received chemotherapy (cisplatin, doxorubicin, or ifosfamide) or denosumab. At the time of the final follow-up data, 3 patients had succumbed to the disease.

### Comprehensive genomic profiling test

**Figure 1:**
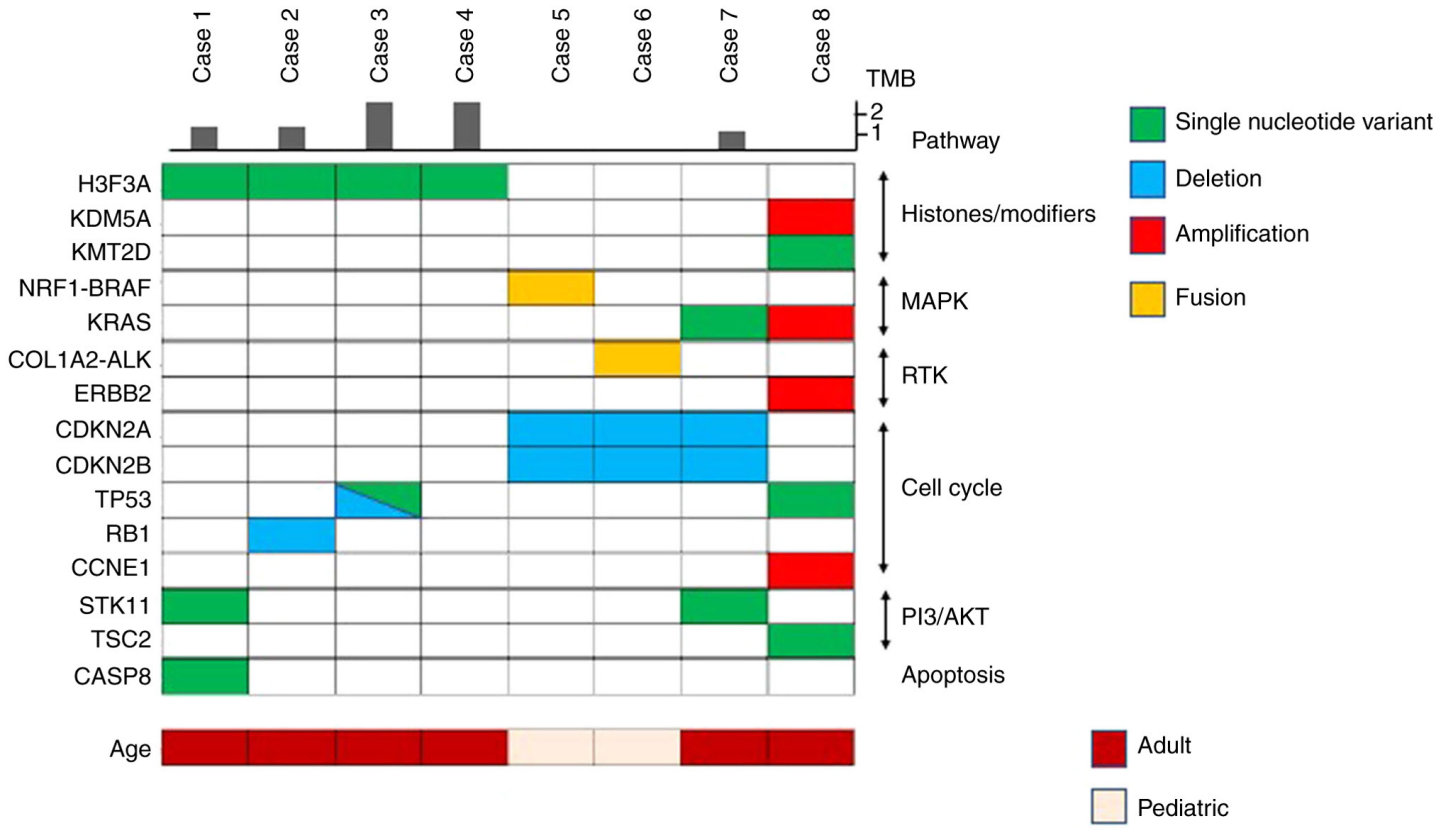
Oncoprint of malignant giant cell tumor of bone. H3F3A, H3 histone family member 3A; NRF1, nuclear respiratory factor 1; COL1A2, collagen type I alpha 2 chain; ERBB2, Erb-B2 receptor tyrosine kinase 2; CDKN2, cyclin-dependent kinase inhibitor 2; CCNE1, cyclin E1; TMB, tumor mutation burden; RTK, receptor tyrosine kinase.

A total of 78 mutations were detected (data not shown). Among these, 26 mutations were annotated as likely or known oncogenic alterations, with an average of 3.1 (26 of 8) alterations per sample (Table II). The oncoprint is depicted in Fig. 1. Single-nucleotide variants accounted for 46% (12 of 26) of the alterations, and copy number alterations (deletion and amplification) and rearrangements (fusion) accounted for 46% (12 of 26) and 8% (2 of 26), respectively. *H3F3A* G34W mutations (hg38, chr1: 226064454 G>T) and G34L mutation (hg38, chr1: 226064454 GG>CT) were found in 3 patients and 1 patient, respectively. In 50% of the cases with *H3F3A* mutation, other co-occurring mutations were related to cell cycle regulators (*TP53* or *RB1*). mTOR pathway gene alterations (*STK11* or *TSC2*) were detected in 3 of the 8 (38%) cases (Fig. 1 and Table II).

In 75% of the cases without *H3F3A* mutation (case nos. 5, 7 and 8; Table II), mitogen-activated protein kinase (MAPK) signaling pathway gene alterations were found (KRAS single nucleotide variant, KRAS amplification, nuclear respiratory factor 1 (*NRF1*)*-BRAF* fusion). Moreover, the collagen type I alpha 2 chain (*COL1A2*)*-ALK* fusion was detected in the remaining one case (case no. 6). All 4 cases without *H3F3A* mutation (case nos. 5-8) had gene alterations related to cell cycle regulators [cyclin-dependent kinase inhibitor 2 (*CDKN2)A* and *CDKN2B* loss, *TP53* mutation and cyclin E1 (*CCNE1*) amplification]. OF note, 1 case had alterations in epigenetic modulator genes, such as *KDM5A* or *KMT2D* (Fig. 1 and Table II).

**Figure 2:**
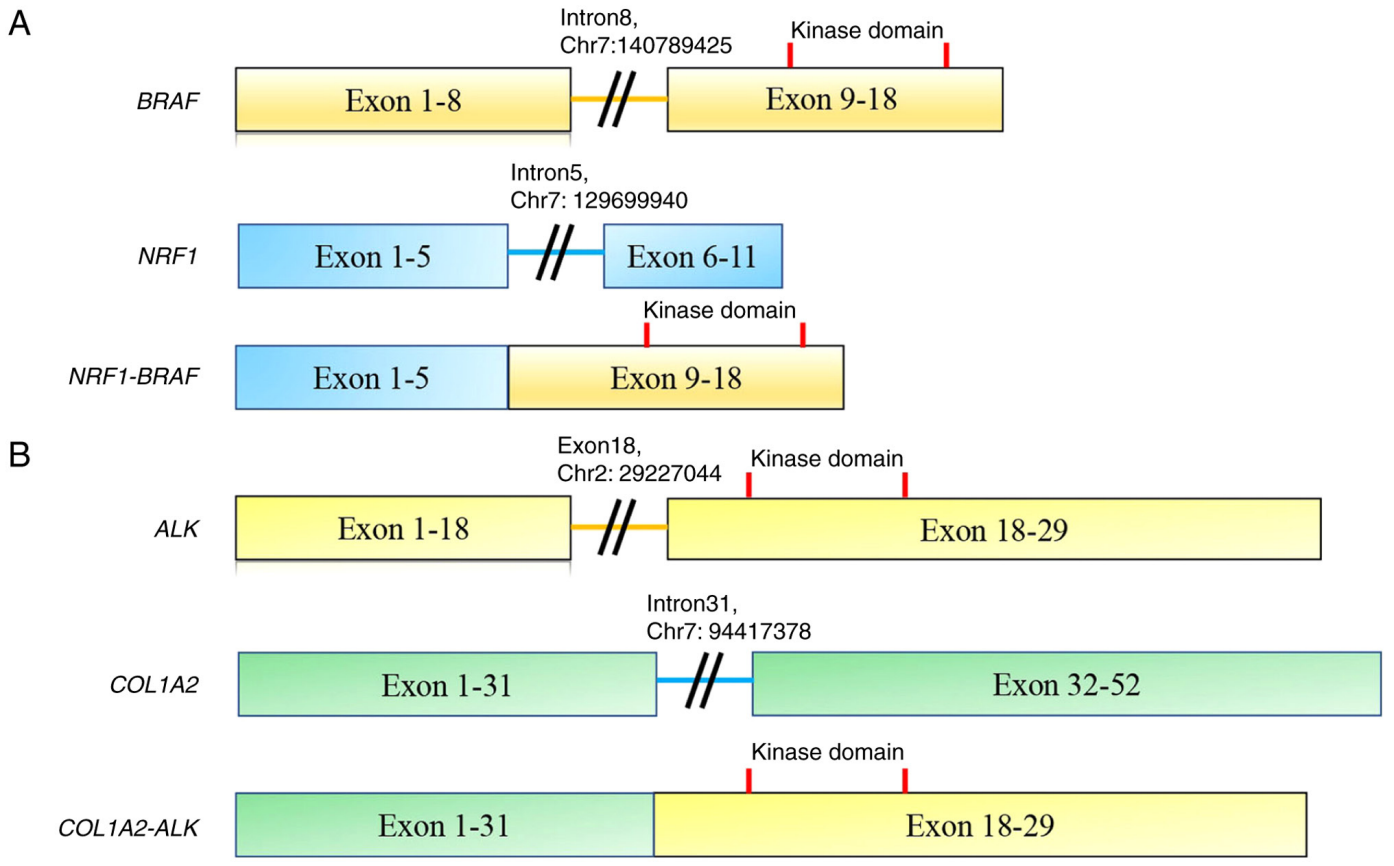
(A) NRF1-BRAF fusion and (B) COL1A2-ALK fusion. NRF1, nuclear respiratory factor 1; COL1A2, collagen type I alpha 2 chain.

**Figure 3:**
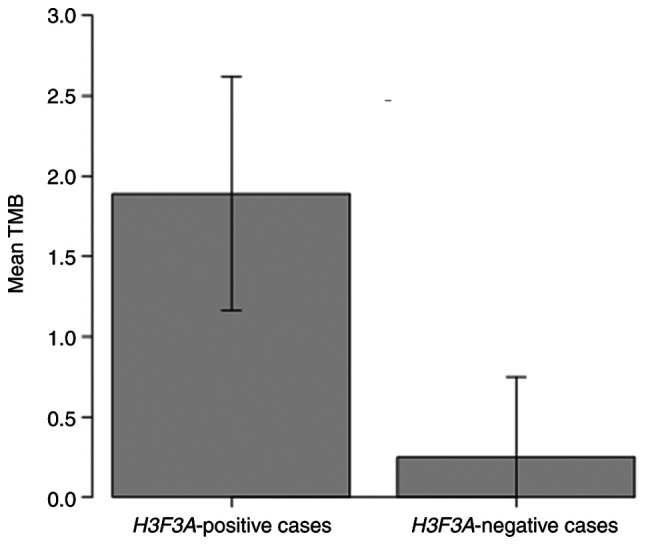
Comparison of the mean TMB between the *H3F3A*-positive cases (case nos. 1, 2, 3, 4) and that of *H3F3A*-negative cases (case nos. 5, 6, 7, 8). The TMB was significantly lower in the samples without *H3F3A* mutation than in the samples with *H3F3A* mutation. Data were analyze using the Student's t-test (mean 0.25 vs. mean 1.89; P=0.01). TMB, tumor mutation burden; H3F3A, H3 histone family member 3A.

*NRF1* intron 5 (chr7: 129699940) was fused with *BRAF* intron 8 (chr7: 140789425) (Fig. 2). The *COL1A2-ALK* rearrangement comprised intron 31 of *COL1A2* (chr7: 94417378) and exon 18 of *ALK* (chr2: 29227044). The kinase domains of both predicted proteins were retained. The tumor mutation burden (TMB) was significantly lower in the samples without *H3F3A* mutation (case 5, 6, 7, 8) than in the samples with *H3F3A* mutation (case 1, 2, 3, 4) (Student's t-test, mean 0.25 vs. mean 1.89, P=0.01, Fig. 3). Of the 8 cases analyzed herein, the patients with kinase fusion had unique characteristics, such as a younger age (9 and 7 years) and a lower TMB (both, 0 muts/Mb) compared to the fusion-negative cases. No patients were enrolled in a trial or off-label use of an approved drug due to trial ineligibility, poor performance status, or unknown reasons.

## Discussion

Using a large genomic database (C-CAT database), the present study analyzed the genomic alterations of clinicopathologically diagnosed malignant GCTB. A total of 4 cases had *H3F3A* mutations and MAPK signaling pathway gene alterations were found in 75% of the cases without *H3F3A* mutation. The most frequent concurrent gene alterations were related to cell cycle regulators, including *TP53*, *RB1*, *CDKN2A/B* and *CCNE1* (75%, 6 of 8 cases). Potentially targetable fusion genes (*NRF1-BRAF* and *COL1A2-ALK*) were also detected.

Malignant GCTB is difficult to characterize due to its rarity, broad histological spectrum and the occasional presence of abundant giant cells in unrelated sarcomas (5). *H3F3A* mutations are detected in benign and malignant GCTB. Although a few *H3F3A* mutation-negative malignant GCTBs have been reported, none have been thoroughly investigated (5). Herein, MAPK signaling pathway alterations were observed in patients with *H3F3A* wild-type tumors. Consistent with these findings, *KRAS* G12V was previously detected in malignant GCTB (8). *HRAS* mutations were also previously found in two cases of malignant GCTB (9), indicating the importance of RAS family mutations in the malignant progression of GCTB. *KRAS* is a frequently mutated oncogene in numerous types of cancer, including non-small cell lung cancer, colorectal cancer and pancreatic ductal adenocarcinoma (14-16). *KRAS* mutations cause conformational changes in *KRAS*-binding Raf proteins, activating downstream effectors involved in cellular growth, differentiation and survival (17).

Cell cycle regulator gene alterations were frequently found in the cohort in the present study. A previous study reported that 80% (4 of 5 cases) of pleomorphic or epithelioid cell-predominant malignant GCTB were positive for TP53 nuclear accumulation (11). Fittall *et al* (10) identified driver events in malignant bone tumors with *H3F3A* mutation using comprehensive genomic and methylation profiling. Malignant progression necessitated additional genetic mutations, such as *TP53* mutations, which was consistent with the findings of the present study. In contrast to the findings of the present study, Fittall *et al* (10) also detected recurrent *TERT* promoter mutation.

The single nucleotide alteration of *H3F3A* induces epigenomic alterations with implications for the development of stromal cells and the tumorigenic process in benign GCTB (18). *H3F3A* mutations are plausibly crucial oncogenic event in malignant GCTB. Other histone modifier gene alterations, such as *KDM5A* or *KMT2D* were detected in the present study, although further studies are required to confirm the importance of these alterations. Biallelic losses of histone lysine demethylase, *KDM4B* or *KDM5A* were previously also found (10). Ishihara *et al* (11) reported that 3 of 4 (75%) cases of spindle cell-predominant malignant GCTBs were negative for H3K27me3 and *EZH2* mutation was found in 1 case, which suggested that the dysfunction of histone methylation, as evidenced by the loss of H3K27me3, may play a key role in the malignant progression of GCTB (11). In contrast to these findings, the *EZH2* mutation was not detected in the present study. The role of the loss of H3K27me3 in malignant GCTB warrants further investigation.

Two fusion genes (*NRF1-BRAF* and *COL1A2-ALK*) need to be carefully validated following the pathology rereview. *BRAF* or *ALK* fusion has not yet been reported in malignant GCTB. The *NRF1-BRAF* fusion gene was previously detected in 2 cases of anaplastic pleomorphic xanthoastrocytoma (PXA) and urothelial carcinoma (19,20). In the case of PXA, the predicted fusion protein contained exons 1-5 of *NRF1* and the serine/threonine kinase domain of *BRAF*. Immunohistochemistry confirmed the robust activation of the MAPK signaling pathway. The loss of *CDKN2A* was also found in the tumor (19). Another case involved a high-grade papillary urothelial carcinoma in the renal pelvis that had invaded the renal parenchyma and spread to the lymph nodes, liver, cervical and lumbar spine and humerus. F1CDx examined a biopsy of the liver lesion and discovered the *NRF1-BRAF* fusion. On the basis of the genomic results, the patient opted to begin a trial of trametinib (Mekinist), a second-generation MEK inhibitor. Following 2.5 months of treatment, an MRI scan revealed that the tumor had shrunk by 48.4% (20). In the present study, in case 5, *NRF1* intron 5 (chr7: 129699940) and *BRAF* intron 8 (chr7: 140789425) were involved, retaining the serine/threonine kinase domain of *BRAF*. Although the confirmation of the fusion transcript and immunohistochemistry for MAPK signaling pathway activation is desirable, the case in the present study may be a candidate for targeted therapy, including MEK and/or *BRAF* inhibitors.

The *COL1A2-ALK* fusion has been found in *ALK*-positive histiocytosis (21). Chang *et al* (21) reported 10 patients with *ALK*-positive histiocytosis, 6 of whom had disseminated disease: A total of 5 cases developed in early infancy with eventual disease resolution, and the 6th patient presented at 2 years of age and succumbed due to intestinal, bone marrow and brain involvement (21). The other 4 patients had localized disease involving the nasal skin, foot, breast and intracranial cavernous sinus; the first 3 patients had no recurrence following surgical resection, and the cavernous sinus lesion resolved completely with the *ALK* inhibitor, crizotinib (21). The association between case 6 in the present study and *ALK*-positive histiocytosis is unknown as the pathology was not rereviewed. Touton-type giant cells have been found in *ALK*-positive histiocytosis (22), which could lead to a misdiagnosis of malignant GCTB. The findings presented herein suggest that potentially targetable *ALK* fusions are present in a subset of cases clinicopathologically diagnosed with malignant GCTB.

The present study has several limitations which should be mentioned. First, the pathology was not rereviewed by a sarcoma pathologist, which may have resulted in some misclassifications. Malignant GCTB in young patients is rare. In particular, two fusion genes should be carefully validated after the pathology re-review by sarcoma pathologists. These two fusion genes may be detected in the resembling tumors, which contain giant cells, apart from malignant giant cell tumor. Second, the C-CAT database lacked the details of fusion gene (in-frame or out-frame). Third, data on whether the tumors were primary or secondary malignant GCTB were not available, and mutation patterns in primary and secondary tumors may differ. However, the real-world data used provide a unique perspective on genomic alterations in clinicopathologically diagnosed malignant GCTB. Fourth, the lack of matched normal control DNA may result in the inclusion of germline mutations inadvertently.

In conclusion, the findings of the present study suggest that MAPK pathway alterations are crucial in *H3F3A*-wild type malignant GCTB. The most frequent oncogenic event was gene alterations related to cell cycle regulators. Potentially targetable *BRAF* or *ALK* fusion may be detected in a subset of cases clinicopathologically diagnosed with malignant GCTB that lack *H3F3A* mutation; however, the careful validation of two fusion genes and a pathology review need to be performed. The real-world findings highlight a unique perspective on genomic alterations in clinicopathologically diagnosed malignant GCTB.

## Figures and Tables

**Table I:** Clinical and genomic characteristics of the patient whose data were analyzed in the present study.

Case no.	Sex	Age, years	*H3F3A* mutation	Metastasis	Drug	Outcome
1	M	35	Mutant	NA	NA	NA
2	F	25	Mutant	Lung, spinal cord, soft-tissue, adrenal grand	CDDP, DOX	NA
3	M	48	Mutant	Lung	CDDP, DOX	NA
4	F	30	Mutant	Lung	CDDP, DOX	DOD
5	F	9	Wild	Peritoneum	No	NA
6	M	7	Wild	No	IFO	Alive
7	M	73	Wild	Bone	Denosumab	DOD
8	M	41	Wild	No	NA	DOD

M, male; F, female; H3F3A, H3 histone family member 3A; NA, not applicable; CDDP, cisplatin; DOX, doxorubicin; IFO, ifosfamide; DOD, died of disease.

**Table II:** Oncogenic alterations identified in the present study.

Case	Gene	Chromosome	Genomic locations	Reference	Base change	Amino acid change	Mutation allele frequency	TMB (Muts/Mb)	MSI
Case 1	CASP8	2	201266689	G	A	R68Q	0.53	1.26	Stable
	H3F3A	1	226064454	G	T	G34W	0.08		
	STK11	19	1223126	C	G	F354L	0.54		
Case 2	RB1	13	48411294-48515183	-	Deletion			1.26	Stable
	H3F3A	1	226064454	GG	CT	G34L	0.41		
Case 3	TP53	17	7673177-7703534	-	Deletion			2.52	
	H3F3A	1	226064454	G	T	G34W	0.20		
	TP53	17	7674241	G	A	S241F	0.07		
Case 4	H3F3A	1	226064454	G	T	G34W	0.14	2.52	Stable
Case 5	CDKN2A	9	21968170-21994454	-	Deletion			0	Stable
	CDKN2B	9	22002171-22010785	-	Deletion				
	NRF1-BRAF	7:7	140789425:129699940	-	Fusion				
Case 6	CDKN2A	9	21968170-21994454	-	Deletion			0	Stable
	CDKN2B	9	22002171-22010785	-	Deletion				
	COL1A2-ALK	2:7	29227044:94417378	-	Fusion				
Case 7	CDKN2A	9	21954945-21998003	-	Deletion			1	Stable
	CDKN2B	9	21998749-22069275	-	Deletion				
	KRAS	12	25245350	C	T	G12D	0.47		
	STK11	19	1223126	C	G	F354L	0.48		
Case 8	CCNE1	19	29763011-29869731	-	Amplification			0	Stable
	ERBB2	17	39651436-39777579	-	Amplification				
	KDM5A	12	285455-389091	-	Amplification				
	KRAS	12	25191796-25295283	-	Amplification				
	KMT2D	12	49050247	TC	T	D1114fs*5	0.05		
	TP53	17	7674903	TTC	T	R209fs*6	0.43		
	TSC2	16	2086815	TTT	T	F1645fs*7	0.14		

TMB, tumor mutation burden; MSI, microsatellite instability; H3F3A, H3 histone family member 3A; NRF1, nuclear respiratory factor 1; COL1A2, collagen type I alpha 2 chain; ERBB2, Erb-B2 receptor tyrosine kinase 2; CDKN2, cyclin-dependent kinase inhibitor 2; CCNE1, cyclin E1.

## Data Availability

The datasets generated and/or analyzed during the current study are available from the corresponding author on reasonable request.
